# Evaluating the Utility of EPIK in a Finger Tapping fMRI Experiment using BOLD Detection and Effective Connectivity

**DOI:** 10.1038/s41598-019-47341-y

**Published:** 2019-07-29

**Authors:** Seong Dae Yun, Ralph Weidner, Peter H. Weiss, N. Jon Shah

**Affiliations:** 1 Institute of Neuroscience and Medicine 4, Medical Imaging Physics, Forschungszentrum Jülich GmbH, Jülich, Germany; 2 Institute of Neuroscience and Medicine 3, Cognitive Neuroscience, Forschungszentrum Jülich GmbH, Jülich, Germany; 3 Institute of Neuroscience and Medicine 11, Molecular Neuroscience and Neuroimaging, JARA, Forschungszentrum Jülich GmbH, Jülich, Germany; 4 JARA - BRAIN - Translational Medicine, Aachen, Germany; 5 Department of Neurology, RWTH Aachen University, Aachen, Germany

**Keywords:** Biological physics, Imaging techniques

## Abstract

EPI with Keyhole (EPIK) is a hybrid imaging technique that overcomes many of the performance disadvantages associated with EPI. Previously, EPIK was shown to provide a higher temporal resolution and fewer image distortions than EPI whilst maintaining comparable performance for the detection of BOLD-based signals. This work carefully examines the putative enhanced sensitivity of EPIK in a typical fMRI setting by using a robust fMRI paradigm – visually guided finger tapping – to demonstrate the advantages of EPIK for fMRI at 3 T. The data acquired were directly compared to the community standard fMRI protocol using single-shot EPI to ascertain a clear comparison. Each sequence was optimised to offer its highest possible spatial resolution for a given set of imaging conditions, i.e., EPIK and EPI achieved an in-planar resolution of 2.08 × 2.08 mm^2^ with 32 slices and 3.13 × 3.13 mm^2^ with 36 slices, respectively. EPIK demonstrated a number of clear improvements, such as superior spatial resolution with favourable robustness against susceptibility artefacts. Both imaging sequences revealed robust activation within primary motor, premotor and visual regions, although significantly higher BOLD amplitudes were detected using EPIK within the primary and supplementary motor areas. Dynamic causal modelling, in combination with Bayesian model selection, identified identical winning models for EPIK and EPI data. Coupling parameters reflecting task-related modulations and the connectivity of fixed connections were comparably robust for both sequences. However, fixed connections from the left motor cortex to the right visual cortex were estimated as being significantly more robust for EPIK data.

## Introduction

Echo planar imaging (EPI)^1^ has been commonly used in functional MRI due to its relatively high temporal resolution. However, because of the fact that EPI realises this high temporal resolution by acquiring multiple lines per excitation, it has a relatively long acquisition window compared to other conventional imaging sequences (e.g. spin-echo or gradient echo). As a consequence, there is more time for phase errors to develop, which in turn leads to potentially severe image distortions.

In order to effectively shorten the acquisition window in EPI, an alternative acquisition strategy, EPI with Keyhole (EPIK), has been presented^2,3^ and validated at 1.5T^4,5^ and 3T^6,7^. The basic principle of EPIK is that since the natural images have most of their energy around the central portion of k-space, this part is fully sampled with the Nyquist criterion, whilst peripheral k-space is sparsely sampled as a single shot of multi-shot EPI. Full sampling of central k-space preserves the image contrast as well as the temporal image SNR with a comparable level to that of single-shot EPI^6,7^. Each EPIK scan is reconstructed by sharing peripheral data from a number of consecutive scans. Here, a sliding window technique is used to ensure that the periphery of k-space is also continually updated, albeit at a slower rate than the keyhole. In this way, a higher temporal resolution and fewer image distortions occur in EPIK, when compared to EPI.

In one of our previous studies^6^, it was shown that EPIK achieved comparable temporal stability and a similar BOLD detection performance to EPI (community standard technique) under the ‘same’ imaging parameters and the ‘same’ image resolution. In a recent study^7^, the capability of higher resolution imaging in EPIK was demonstrated with a high-resolution visual fMRI study. The study also showed that potential autocorrelation effects from the sharing of peripheral data in EPIK were too small to have an impact on the analysis of time-series data.

In contrast, the present work focuses on the investigation of the putative advantages of EPIK for fMRI. For this purpose, visually guided finger-tapping fMRI was performed to carefully examine brain activations in the visual and motor regions. Furthermore, in addition to the analyses of BOLD amplitudes, performance differences of EPIK and EPI were evaluated with regard to effective connectivity analyses between brain regions using dynamic causal modelling (DCM)^8^.

The EPIK sequence was optimised in a way that it achieved its highest possible image matrix size and number of slices whilst the other imaging parameters were kept identical (e.g. FOV, TR/TE, etc.). The optimised EPIK sequence was employed in a direct head-to-head comparison with single-shot EPI, which was also optimised in the same way. Consequently, the performance of EPIK was evaluated against single-shot EPI in the same functional experiment using a standard fMRI protocol. Previously, numerous studies have adopted acceleration techniques such as parallel imaging^9,10^, partial Fourier^11,12^ or multi-band techniques^13^ to enhance the spatial or temporal resolution in fMRI^13–16^. However, during the sequence optimisation in the present study, the use of acceleration techniques was excluded in order to avoid the deleterious effects thereof on the performance evaluation of each imaging method.

The parameters of interest were i) BOLD signal amplitude in motor and visual areas and ii) the modulation parameters, estimated by DCM, involved in the visually guided finger-tapping task. Based on the sequence characteristics of EPIK, we hypothesise that the performance of EPIK will be demonstrably better than standard EPI with regard to BOLD amplitude. That is to say, in a given TR, we expect EPIK to produce images with a higher spatial resolution and to be more immune to signal dropout. In particular, the higher spatial resolution achieved by EPIK is expected to result in a more accurate localisation of BOLD signals from activated voxels and to minimise the impact of non-activated neighbouring regions that would reduce the observed BOLD amplitude. Furthermore, given the higher spatial resolution, it might then be possible to measure the functional network dynamics within a given network of brain areas more reliably. Accordingly, DCM on EPIK and EPI are expected to identify the same winning models, since the observed network dynamics should per se be identical. However, we hypothesise that the coupling parameters estimated on the basis of EPIK data may be more robust across different scanning sessions.

## Materials and Methods

### Pulse sequence: EPIK

**Figure 1:**
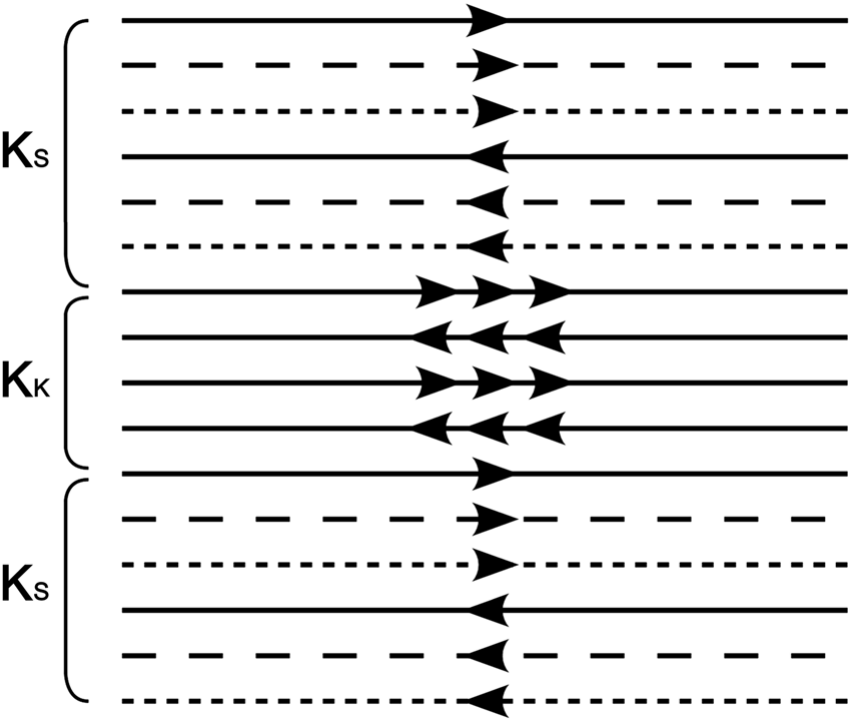
Schematic representation of the k-space trajectory of three-shot EPIK. The k-space trajectory consists of full Nyquist sampling for the central k-space (Keyhole: K_K_) and segmented, interleaved acquisition for peripheral k-space (K_S_). Similar to three-shot EPI, for the peripheral k-space, every third line is sampled at each scan. The first line number taken at the 1st, 2nd and 3rd scan is 1, 2 and 3, as denoted with solid, dashed and fine-dashed lines in the figure. The first line number becomes 1 again at the 4th scan.

EPIK combines the acquisition of a central portion of k-space – the “keyhole” – with a continuous, interleaved update of the high spatial-frequency information from the periphery of the k-space. In stark contrast to the original keyhole method, the continuous update of the high spatial-frequencies ensures that only small parts of the k-space periphery are correlated and those only in a limited number of scans. Figure 1 shows the schematic representation of the k-space trajectory for three EPIK scans. Each scan comprises a central keyhole and an interleaved acquisition of the peripheral lines of k-space. Here, the central k-space region (k-space keyhole: K_K_) is completely sampled with the Nyquist criterion (Δk_y_ = 1/FOV), whilst the peripheral k-space regions (k-space sparse: K_S_) are sparsely sampled with Δk_y_’ = 3/FOV. The first line number of the peripheral k-space at the 1st, 2nd and 3rd scan is 1, 2 and 3, respectively; the first line number becomes 1 again at the 4th scan. Through the sharing of the sparse region data originating from three consecutive scans, and with the keyhole region being updated for every measurement, complete k-space data can be obtained for every scan. Effectively, then, K_K_ is updated after every scan and K_S_ is updated every fourth scan. To ensure that the phase increases smoothly when sharing the sparse region data, correct echo time shifting (ETS)^17^ is an integral part of the sequence. In this study, the keyhole region was configured as one-fourth of the k-space and thus, the total number of phase encoding lines to be sampled in each EPIK scan reduces to half of that for comparable EPI. When a fourth scan is acquired, the peripheral k-space data from the first scan are discarded and thus the periphery, K_S_, is updated by application of this sliding window.

For this work, the above configuration was employed on a Magnetom Tim^®^ Trio 3 T MRI scanner (Siemens Medical Solutions, Erlangen, Germany) with a 12-channel phased array coil from the manufacturer.

### Point spread function (PSF)

For each imaging method (i.e. EPI and EPIK), the signal decay trajectory was simulated as a function of the sampled k-space line and the corresponding PSF was obtained. Here, the T_2_^*^ value used in this simulation was 66 ms, which is known to be the T_2_^*^ value of the grey matter at 3 T^18,19^. From the PSF plot of each method, the full-width at half-maximum (FWHM) was examined and compared between the imaging methods.

The performance of EPIK in PSF was also evaluated under the conditions of fMRI. The very same simulation described above was carried out for the case when there were dynamic signal changes. Here, the rate of dynamic signal changes was set to 5% from the 1st to the 3rd scan. The 5% change is a typical ratio of BOLD signal increase relative to the baseline at 3 T. The purpose of using a rapid signal variation here (i.e. 5% change within 3 temporal scans) was to simulate the worst case in EPIK. Note that in reality, the dynamic signal changes from the 1st to 3rd scan is not as rapid as in the current simulation.

### fMRI experiments

A visually-guided finger-tapping paradigm was employed for *in vivo* fMRI experiments to elicit robust activations in visual and motor brain regions. Each experimental session started with a 20 s baseline period (fixation) followed by 7 periods of 1 Hz finger tapping with the right index finger (duration 32 s) triggered by a visual stimulus (two red lines centrally presented above and below the black fixation cross, which was displayed throughout the entire duration of the measurement). The finger tapping activation blocks were alternated with resting blocks of a duration of 32 s, during which the subject did not move, but continued to fixate on the black fixation cross. Scanning comprised 2 dummy scans in order to a reach steady state. Thereafter, 225 scans were acquired resulting in a total acquisition time of 8 min 29 s for each fMRI run. A single healthy male volunteer, screened to exclude neurological and psychiatric illnesses, participated in the study. After a complete description of the study, written informed consent was obtained prior to scanning. The local institutional review board (IRB; RWTH Aachen University, Germany) approved the study protocol, screening questionnaires and consent forms. All experiments were performed in accordance with relevant guidelines and regulations.

Functional data acquisition was carried out on a weekly basis on the same subject. For each session, fMRI data were acquired with the following parameters for both sequences: FOV = 200 × 200 mm^2^, flip angle = 90°, TR/TE = 2200/30 ms and slice thickness = 3 mm with a distance factor of 10%. Under the given imaging conditions above, the voxel size/number of slices achieved by EPI and EPIK were 3.13 × 3.13 mm^2^ (matrix: 64 × 64)/36 and 2.08 × 2.08 mm^2^ (matrix: 96 × 96)/32, respectively. The fMRI runs were repeated twice using the two imaging methods, EPI and EPIK, in alternating order. Thus, of the 14 sessions acquired in total, 7 sessions started with EPI (followed by EPIK) and 7 started with EPIK (followed by EPI). This was to control for possible habituation effects (potentially leading to a decrease of the blood-oxygenated-level-dependent (BOLD) response) in this robust, but simple, finger-tapping paradigm. It is noted that EPIK employs data sharing and therefore it is true that a single EPIK volume contains data from three TRs whereby the keyhole, which has over 90% of the SNR, is unique to each of the three shots. The TRs required to reconstruct the EPIK volumes is three times TR (i.e. 6600 ms). However, since a sliding window reconstruction is applied for EPIK, the temporal throughput is also the same as EPI (i.e. one EPIK temporal volume every 2200 ms).

### Analysis of fMRI data

For all 14 sessions, the two fMRI runs based on EPI and EPIK were separately analysed. The statistical parametric mapping software SPM12 (Wellcome Department of Imaging Neuroscience, London; http://www.fil.ion.ucl.ac.uk/spm/software/spm12/) was used for preprocessing and analysis of the functional imaging data. Functional images were spatially realigned to correct for interscan movements. Then, the mean EPI/EPIK image of each session was computed and spatially normalised to the MNI single-subject template using the normalisation function as implemented in SPM12. Finally, data were smoothed using a Gaussian kernel of 4 mm full-width half-maximum.

On the first level, an onset regressor defined the onsets of finger tapping blocks, and block length was set to 32 s for each session. The haemodynamic response was modelled using a canonical haemodynamic response function (HRF) and its time derivative. The six head movement parameters, as estimated during the realignment procedure, were included as explanatory regressors in the model. To specify the first-level contrasts, each regressor of interest was compared with the implicit baseline by setting this regressor to 1 and all other regressors to zero. The resulting contrast images were then entered into a second level analysis.

On the second level, model estimation was performed using the ANOVA flexible factorial design as implemented in SPM12, with sessions as a covariate to remove between-session variance. To test the conjunction null hypothesis, a conjunction analysis was performed on the baseline contrasts to identify voxels that were significantly activated in both the EPI as well as in the EPIK sessions. Furthermore, BOLD amplitudes, as measured by EPIK and EPI, were compared within these voxels.

### Dynamic causal modelling (DCM)

In order to compare the effects of EPIK relative to EPI on effective connectivity, dynamic causal modelling (DCM)^8^ was implemented separately for both sets of data. DCM demands slice-time corrected data. Accordingly, for the DCM analysis, data pre-processing was repeated but including slice-time correction as implemented in SPM12. All other pre-processing parameters and steps were identical, as described above.

The task performed in the present study was a visually guided finger-tapping task that requires an information exchange between the visual and the motor systems. Hence, the effective connectivity pattern between early visual areas and the primary motor area was particularly relevant. Three volumes of interest (VOIs) were defined based on the MNI coordinates obtained in the standard GLM second-level analyses and the participant’s individual anatomy. Visual information was presented centrally in the visual field and was hence represented bilaterally in visual cortex. Therefore, two VOIs were selected, one in each hemisphere. Centre coordinates for each visual VOI were chosen based on the activation pattern observed in the second-level conjunction analysis. In particular, in each hemisphere, the coordinates of the maximum activated voxel within the largest cluster found in the occipital cortex were set as the centre of the VOI. Accordingly, MNI coordinates for the left visual VOI were MNI [−32 −88 6] and those for the right visual VOI were MNI [36 −78 −2]. In order to derive the coordinates for the VOI representing the primary motor area M1, a two-step procedure was implemented. First, based on the subject’s anatomical image, the hand knob contralateral to the response hand was identified. In a second step, the activation pattern as observed in the second-level conjunction analysis was taken into account. The nearest local maximum activation with regard to the hand knob was defined as the centre VOI and was located at MNI [−30 −26 58].

For each session, the first eigenvariate was extracted from voxels within 4 mm sphere centred at the respective MNI coordinate (exceeding a threshold of 0.8). Accordingly, three signal time courses were extracted for each session and for each sequence.

**Figure 2:**
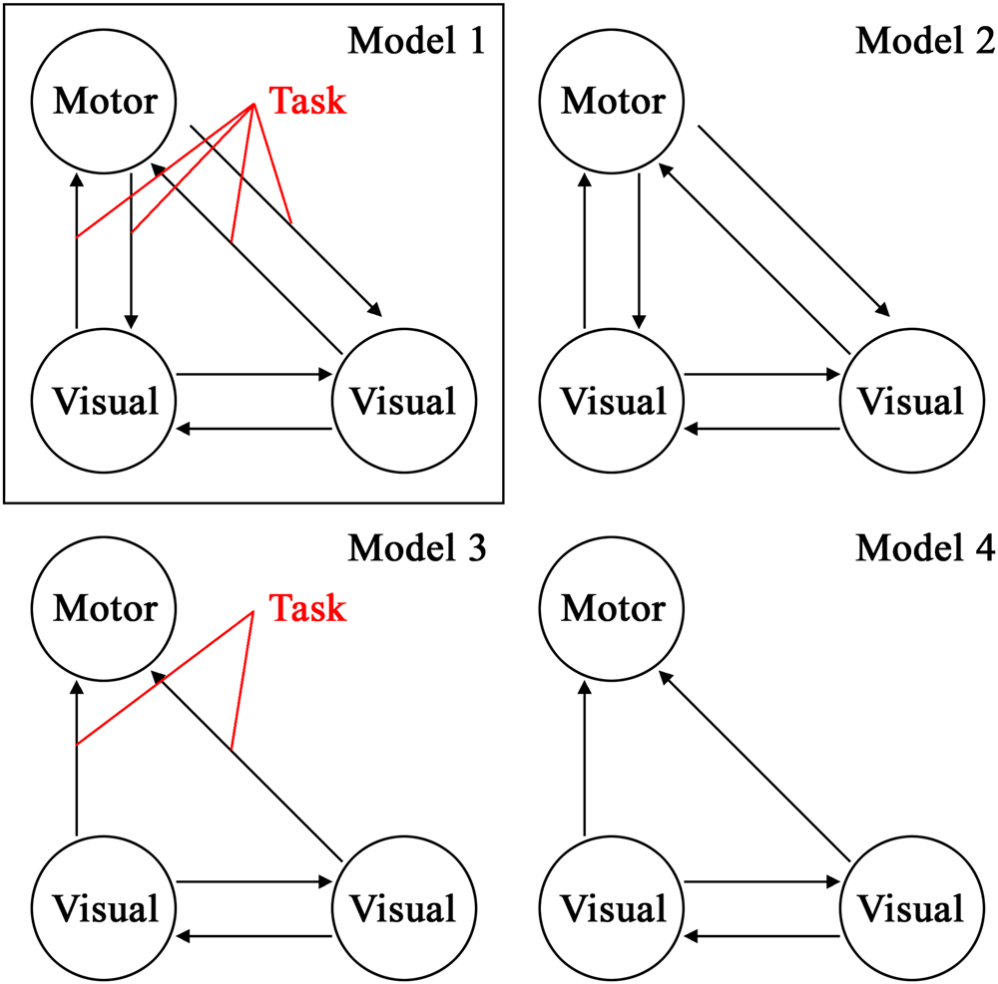
Model space consisting of all DCM model tested. Three volumes of interest (VOI) were included in the model, involving the left motor cortex and the early visual regions in both hemispheres. Reciprocal connections between both visual regions were constant in all models. Models 1 and 2 had bi-directional connections between the motor and visual regions. Models 3 and 4 only involved forward connections from the visual to the motor cortex. Task related modulations of connections between the visual and the motor areas were included in Models 1 and 3. For both EPI and EPIK data, Bayesian model selection (BMS) identified Model 1 as the winning model.

Four different DCM models (Fig. 2) were defined, taking into account signal time courses from these three regions, and hence constituted the model space for the subsequent Bayesian model selection^20,21^, which allows the most likely DCM to be identified. All models involved bidirectional fixed connections between both visual areas. Two models involved bidirectional fixed connections between both the visual areas and the motor area (models 1 and 2). In one of these models (model 1), connections between the visual and the hand areas were modulated by the experimental task. Two additional models (models 3 and 4) comprised unidirectional connections from both visual areas to the hand area, rather than bidirectional connections. In model 3, the connection from the visual area to the motor area was modulated by the experimental task, while no such modulation was implemented in model 4. For all models, the experimental task was set as the driving input for the given DCMs via both visual regions.

These four DCM models were estimated separately for the EPIK and the EPI sessions using DCM12 as implemented in SPM12. A random-effects Bayesian model selection was performed separately for both data sets to identify the most likely DCM^21^.

Furthermore, the variability of coupling parameters of the winning model was compared between EPIK and EPI. Therefore, the variance of the estimated DCM parameters reflecting directional intrinsic connections in the model (A-matrix) was estimated separately for each imaging sequence and then compared using an F-test. Similarly, the variance of parameters reflecting task-induced connectivity modulations (B-matrix) were estimated and compared via F-tests.

## Results

### Reconstructed images

**Figure 3:**
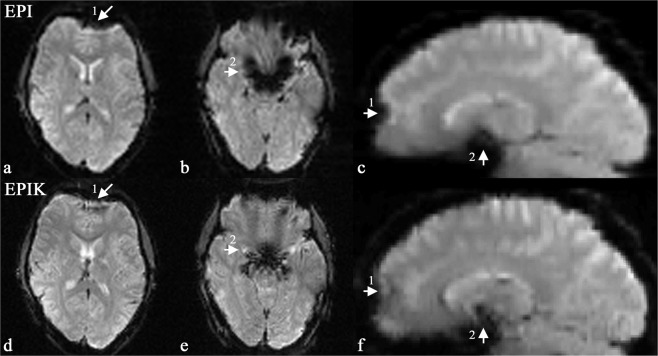
Reconstructed images of the brain of a healthy human volunteer acquired with EPI (64 × 64: 3.13 × 3.13 mm^2^) and EPIK (96 × 96: 2.08 × 2.08 mm^2^). Fig. a and b show two slices from EPI and Fig. d and e show the corresponding slices from EPIK. Fig. c and f display the sagittal plot of EPI and EPIK slices. This example depicts how the EPIK images exhibit reduced susceptibility artefacts compared with the EPI images, particularly around in the region of the sinuses (marked by arrow 1) and in the region of the fourth ventricle (marked by arrow 2).

Figure 3 shows reconstructed *in vivo* images from a representative fMRI session. Two slices were chosen out of the multi-slice data set to best demonstrate the reduced susceptibility artefacts in the data obtained with EPIK. For comparison between EPI and EPIK, each slice was taken at a nearly identical slice position. Visual inspection of the figure suggests that the EPIK image (matrix size: 96 × 96; in-plane pixel size: 2.08 × 2.08 mm^2^; see Fig. 3d,e) were reconstructed without any significant loss of signal or any severe degradation of image quality and, furthermore, the slices exhibit a noticeably enhanced spatial resolution, when compared with the EPI images (matrix size: 64 × 64; in-plane pixel size: 3.13 × 3.13 mm^2^; see Fig. 3a,b). Moreover, due to the reduced number of lines per shot in EPIK and the use of an effectively shorter readout per phase encoding line in EPIK (0.445 ms) than in EPI (0.510 ms), a substantial reduction of geometric distortions was observed in the EPIK images. Signal drop-out due to susceptibility differences is easily visible in the EPI images, particularly in the region of the sinuses (marked by arrow 1 in Fig. 3a,c) and in the region of fourth ventricle (marked by arrow 2 in Fig. 3b,c); this signal dropout is more circumscribed in the EPIK images. To further aid comparison, the reconstructed axial slices are also presented in sagittal orientation (see Fig. 3c,f), which clearly reveals the reduction of geometric distortions in EPIK for the regions marked by both arrows.

### Point spread function (PSF)

**Figure 4:**
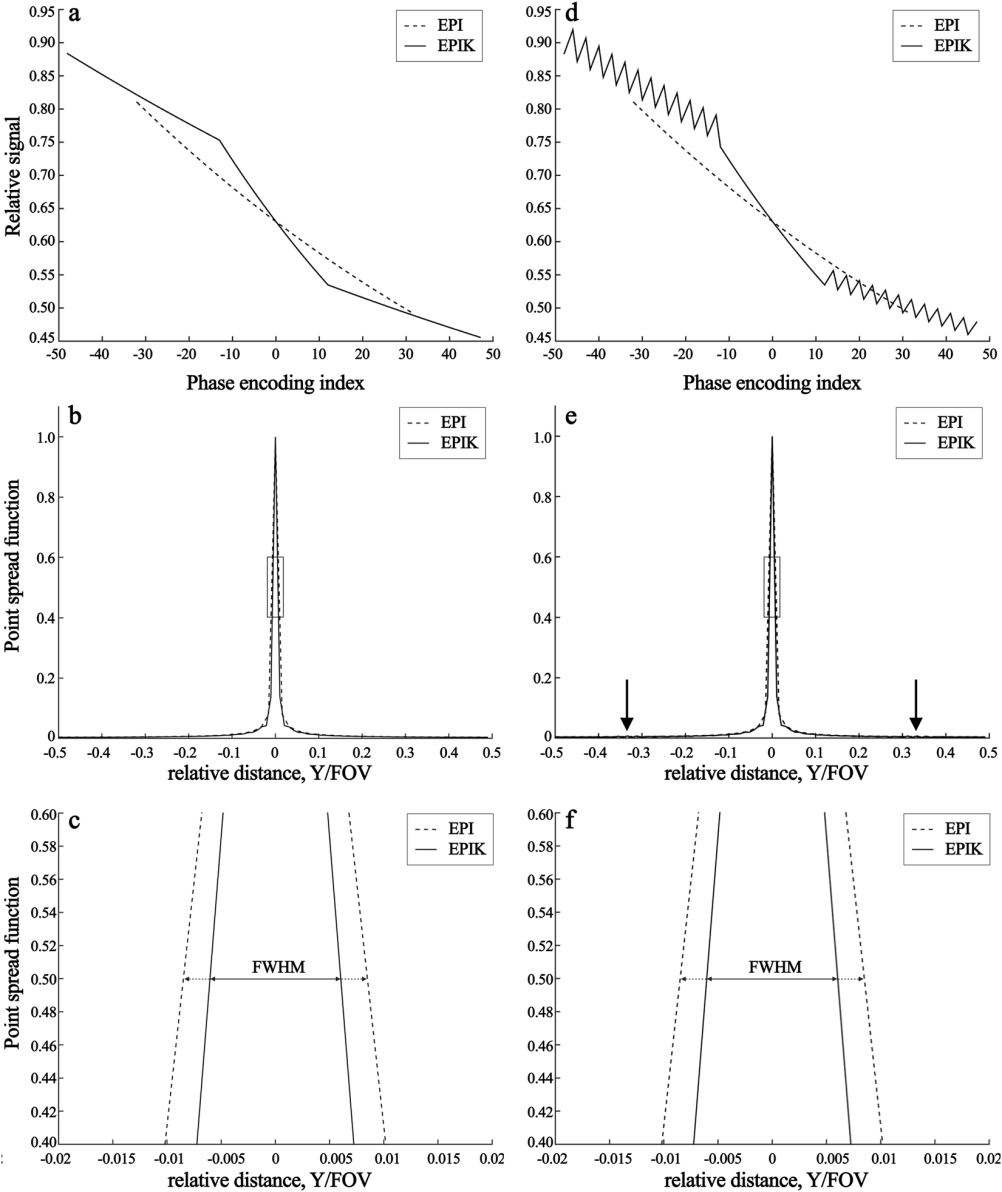
(**a**) Signal decay trajectories of EPI and EPIK with respect to the phase encoding index, (**b**) magnitude PSFs for the entire range and (**c**) for the central region (marked by the square in (b)) and (**d**–**f**) results obtained from the same analysis of (a-c), but with the condition of dynamic signal changes: 5% from the 1st to the 3rd scan. The dotted and solid lines indicate EPI and EPIK, respectively.

Figures 4a and b depict the signal decay trajectory and the corresponding PSF for the stationary case (i.e. no dynamic signal changes). The FWHM of EPI (0.0170) was larger than that of EPIK (0.0121), which can be also visually verified from the enlarged plot of the central region (Fig. 4c). The smaller FWHM in EPIK implies that image blurring caused by the long echo train is less pronounced in EPIK than in EPI, i.e. EPIK has a better spatial resolution than EPI. For the present study, where the applied FOV was 200 mm, the FWHM in a mm-scale was 3.40 mm and 2.41 mm for EPI and EPIK, respectively, that is, an improvement of almost 41% for EPIK.

Figures 4d and e show the results obtained on the setting with the dynamic signal changes. As shown in Fig. 4d, signal fluctuations are presented for the peripheral k-space regions, which were caused by the dynamic signal changes from the 1st to the 3rd scan. These signal fluctuations created two small peaks in the PSF, each of which is located in the tails of each side (see the arrows in Fig. 4e). However, the magnitude of these peaks is very small (0.0068) and thus is expected to have little impact on image quality. The FWHM of EPIK was slightly increased as compared to the stationary case. However, its increase was only 0.16%, which is fairly negligible.

### Sequence-specific differences in brain activation patterns

In order to assess both the common and differential activation patterns elicited by the two imaging sequences, the first level baseline contrasts were calculated for each session and were then taken to the second level. On the second level, a flexible factorial design, as implemented in SPM12, was set up, with “sessions” as covariates to remove between-session variance. A conjunction analysis was performed testing the conjunction null hypothesis for significant effects in both imaging sequences. The identified regions are listed in Table 1 with the respective statistical quantities such as cluster size, MNI-coordinates and t-scores. The statistical threshold for activated regions was set to a corrected p-value < 0.05 (FWE at the cluster-level based on an uncorrected threshold of 0.001 at the voxel-level).

**Table 1:** Brain regions whose activity has consistently been detected by EPIK and EPI.

Brain Region	Cluster size	Side	MNI-Coordinates			T-score
Middle/inferior occipital gyrus	1344	L	−32	−88	6	18.51
Inferior occipital gyrus/Cerebellum	3310	R	36	−78	−2	17.73
Superior temporal gyrus/Rolandic operculum	549	L	−40	−34	24	17.41
Precentral gyrus	143	L	−58	4	24	17.38
Precentral gyrus/Middle frontal gyrus /Superior frontal gyrus/Postcentral gyrus	321	R	48	0	54	17.26
Pre/postcentral gyrus	2379	L	−28	−38	56	17.00
Superior temporal gyrus/Supramarginal	293	R	50	−28	24	15.72
Medial frontal cortex/SMA	881	L	−2	−4	70	15.48
Rolandic operculum	156	L	−42	−4	12	12.46
Superior parietal cortex	58	L	−22	−50	50	11.25
Middle temporal gyrus/Angular gyrus	106	L	−54	−56	14	11.04
Right precentral gyrus	48	R	54	0	22	10.26
Cerebellum	160	L	−28	−62	−18	9.26
Superior occipital sulcus	49	L	−24	−72	22	9.21
Putamen	67	L	−22	−2	6	7.14
Thalamus	60	L	−12	−20	10	5.06

For the conjunction analysis “visually-guided finger tapping” versus “baseline”, both imaging sequences consistently revealed significant activations within large networks comprising both motor and visual brain regions. In particular, strong activation was found in the left primary motor cortex (M1), including the hand representation contralateral to the moving right hand. In addition, significant BOLD signals were observed in the medial (and lateral) frontal regions including the supplementary motor area (SMA) and the premotor cortex (PM). Bilateral cerebellar activations were also observed and were clearly stronger in the right cerebellar hemisphere, i.e., ipsilateral to the right moving hand. Motor activation was complemented by significant signal changes in early and higher visual areas (Table 1).

**Figure 5:**
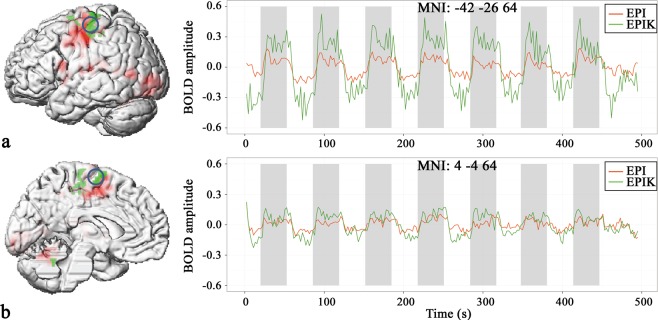
(**a**) The time courses from the maximally activated voxel (−42, −26, 64) within the primary motor cortex (M1, core region of the finger tapping network) and (**b**) the time courses of a representative voxel (4, −4, 64) in the supplementary motor area (SMA). On the left of each sub-figure, the location of the MNI coordinate is indicated by the blue circle overlaid on the brain.

Within a subset of these brain regions, EPIK yielded significantly stronger BOLD amplitudes in a number voxels as compared to EPI (p < 0.05, FWE at the cluster-level based on an uncorrected threshold of 0.001 at the voxel-level). Stronger activation was found with EPIK within the core regions of our finger-tapping network. These activations were located in the left postcentral gyrus, extending to the precentral regions involving primary motor regions such as areas 4 a and p^22^. In addition, EPIK revealed stronger BOLD amplitudes in supplementary motor areas (SMA), the right cerebellum as well as in right visual cortex (Fig. 5, Table 2). The reverse contrast on voxels of the finger-tapping network (EPI > EPIK) did not reveal any significant supra-threshold voxels.

**Table 2:** Brain regions that show higher activity when measured with EPIK as compared to EPI.

Brain Region	Cluster size	Side	MNI-Coordinates			T-score
Pre-/Postcentral gyrus	132	L	−42	−26	64	7.56
Medial frontal cortex/SMA	82	R	4	−4	64	6.66
Inferior/Middle occipital gyrus	39	R	36	−78	−2	7.04
Cerebellum	32	R	20	−48	−28	6.37
Cerebellum	29	R	6	−58	−24	6.94

Labelling of the anatomical location was based on the Anatomy toolbox implemented in SPM^23^. To further demonstrate the different sensitivities of EPIK and EPI in these regions, signal time courses were extracted from the voxel in the primary motor area (Brodmann area (BA) 4a) exhibiting the most marked statistical difference (MNI: −42, 26, 64; T = 7.56). On average, “visually-guided finger tapping” induced a signal increase of 10.3% relative to the “baseline” when measured with EPI. In contrast, when measured with EPIK, the same analysis revealed a 36% increase in BOLD signal, corresponding to a signal increase by a factor of 3.5 (Fig. 5a).

### DCM analyses: Sequence-specific differences in brain functional connectivity

Random-effects Bayesian model selection revealed model 1 as being superior to all other models, as indicated by a protected exceedance probability^24^ of 0.9471 compared to those from other models: 0.0072 (model 2), 0.0407 (model 3) and 0.0049 (model 4) in EPIK; the probabilities from EPI were 0.9957 (model 1), 1.5217e-04 (model 2), 0.0041 (model 3) and 5.2191e-05 (model 4). Hence, a bidirectional model, involving task-related modulations of connection between visual and motor areas, can be considered the best model. This finding has been confirmed by both the EPI, as well as the EPIK data.

In the next step, the accuracy with which the parameters were estimated was compared between the EPI and EPIK sessions. This was performed separately for every fixed connection (A-matrix) as well as for task-related changes in effective connectivity (B-matrix). In particular, using an F-test, it was tested whether the variance of DCM parameters across different scanning sessions was significantly smaller when data were acquired with EPIK as compared to EPI. The coupling parameters of the intrinsic connection between the hand area and the right visual area had a significantly smaller variance for EPIK compared to EPI (F(13,13) = 4.8796 p > 0.05, Bonferroni corrected for multiple comparisons). For all other connections, no difference in variance was observed (Table 3, all F-scores larger than F(13,13) = 0.45834 and all p-scores approximated a value of 1). Similarly, for task-related changes in connectivity as reflected by B-matrix parameters, no significant differences in variance were observed (Table 3, all F-scores larger than F(13,13) = 2.7952 and all p-values larger than p = 0.22).

**Table 3:** F-scores and p-values testing for lower variance in EPIK compared to EPI.

Brain Region	Hand	Visual left	Visual right
**Hand**	F(13,13) = 0.68787 p = 1 n.s.	F(13,13) = 0.49456 p = 1 n.s.	F(13,13) = 1.4384 p = 1 n.s.
**Visual left**	F(13,13) = 0.54103 p = 1 n.s.	F(13,13) = 0.45834 p = 1 n.s.	F(13,13) = 1.11 p = 1 n.s.
**Visual right**	**F(13,13) = 4.8796 p = 0.033** *	F(13,13) = 0.71235 p = 1 n.s.	F(13,13) = 0.91628 p = 1 n.s.

Tests were performed separately for the connectivity parameters of the fixed connections in the DCM-model (as given by the DCM A-matrix). Connection directionality is indicated from rows to columns, i.e. row labels indicate a connections source region, whereas column labels indicate the target brain region. The connection with a significant lower variance for EPIK is highlighted in bold. All p-values are corrected for multiple comparisons using a Bonferroni correction.

## Discussion

The work presented here demonstrates the utility of the EPIK sequence in a visually-guided finger-tapping task at 3 T. The reconstructed images from a representative fMRI session verify that EPIK outperforms the standard functional sequence, EPI, in terms of imaging resolution and robustness against geometric distortions. The functional data also show that EPIK identified stronger BOLD amplitudes than EPI. Moreover, effective connectivity analyses using DCM indicate a similar performance of EPIK and EPI with regard to parameters that reflect task-induced connectivity changes between brain regions. However, coupling parameters describing intrinsic connections from the left motor to the right visual cortex were more reliably estimated with EPIK.

The imaging parameters of the two sequences (e.g. EPI and EPIK) were adjusted in a way that the maximum possible matrix size and the number of slices were used under the condition that the same FOV, TR and TE were applied without any acceleration techniques; a matrix size of 96 × 96 with 32 slices and a matrix size of 64 × 64 with 36 slices were achieved by EPIK and EPI, respectively. We also noted that if parameters such as TR are changed to facilitate a better spatial resolution of EPI, the same change will lead to an even better resolution of EPIK; that is, EPIK stays a step ahead. It is further noted that the smaller number of slices acquired in EPIK was a direct consequence of the improved spatial resolution. Nevertheless, the acquired 32 slices (with a slice thickness of 3 mm – as for EPI) were clearly sufficient to assess all the relevant brain regions involved in the visually-guided finger tapping task, since the EPIK sequence covered almost the whole brain from the parietal cortex to the cerebellum (see Results). In adjusting the sequence protocols, acceleration techniques such as parallel imaging or partial Fourier were not employed in this study. This was done to avoid the deleterious effects thereof on the evaluation of the sequence performance, as the use of acceleration techniques would have made it more difficult to unambiguously attribute advantages and disadvantages to each sequence. Despite the more complicated reconstruction scheme, EPIK images were reconstructed without any degradation of image quality and exhibited enhanced spatial resolution compared to those from EPI.

Essentially, EPIK shortens the effective readout time per k-space traversal by only applying the multi-shot scheme for the periphery of k-space. For the reconstruction of EPIK images, the peripheral k-space regions are completed by sharing the data from neighbouring scans. This repeated use of the k-space periphery may introduce an autocorrelation. However, in EPIK, only small parts of the k-space periphery are correlated in a limited number of scans. Importantly, this correlation is only given for the high spatial frequencies containing edge information. In contrast, keyhole data are unique to each shot. The feasibility of using EPIK for fMRI at 3 T has been previously demonstrated in our earlier work^6^ showing that the temporal stability of EPIK, represented in temporal signal-to-noise ratio (tSNR), was comparable to that of EPI, and thus detection of BOLD responses was not significantly reduced for EPIK. Furthermore, the capability of EPIK in tracking the dynamic BOLD signal changes was investigated with actual functional data (16-subject data) as well as the BOLD simulations using the established MRI simulator, JEMRIS (Jülich Extensible MRI Simulator)^25^. Indeed, the simulation results demonstrated that the signal behaviour of the ‘BOLD’ response, as reproduced by EPIK, showed a higher degree of agreement with the input vector than did EPI. In addition, the autocorrelation issue has also been thoroughly investigated in our recent study^7^. In that study, it was shown that the autocorrelation effect induced from the EPIK reconstruction scheme does not have a significant impact on the detection of BOLD signals. Moreover, it has also been shown that the comparable multi-shot EPI method (i.e. three-shot EPI) had a substantially reduced performance in detecting the BOLD signals when compared to single-shot EPI and EPIK^6,7^. This was mostly due to the fact that the central k-space is also sparsely sampled in multi-shot EPI, leading to a considerable magnification of physiological noise resulting from the shot-to-shot instabilities. As a result, multi-shot EPI has been shown to have a significantly reduced tSNR. Therefore, in order to be feasible for use in dynamic MR applications, multi-shot EPI methods require additional correction for physiological noise.

In this work, a functional study with a visually-guided finger-tapping task has been performed using the EPIK sequence in direct comparison with a single-shot EPI sequence that is commonly used as the standard protocol for fMRI. The functional results obtained show that stronger sensitivity in the detection of BOLD activation was observed in EPIK. This was observed in the area involving the primary motor, as well as the supplementary motor cortex. The stronger activation was mostly due to the reduction of the partial volume effects in EPIK arising from the increased image resolution^26^. More specifically, a higher image resolution allows a more specific localisation of BOLD signals from activated voxels by minimising artefacts from neighbouring, non-activated regions. The actual echo spacing of each phase encoding line in EPIK was 0.890 ms. However, due to smaller number of readout lines in each shot of the EPIK scheme, the effective echo spacing required for each phase encoding line is reduced to half, which is 0.445 ms (i.e. 0.890/2); that is, the matrix size was 96 and each shot acquired 48 lines. This is shorter than that of EPI (0.510 ms) and as a consequence, it was demonstrated that EPIK was less prone to the susceptibility artefacts existing in the prefrontal area and fourth ventricle as shown in Fig. 3. As a consequence, the recovered signals (i.e. reduced susceptibility artefacts) in such an area can have a contribution to the stronger BOLD activations in a related fMRI study probing those regions.

The benefits of increased sensitivity in detecting BOLD activation and the higher spatial resolution not only affects the standard BOLD amplitude analyses but may also be beneficial for modelling approaches based on BOLD signals. DCM infers neural states and interactions between brain regions on the basis of measured BOLD signals. Increased image resolution and a less distorted, and hence more robust signal, due to reduced partial volume effects may allow a more reliable and efficient estimation of model parameters. This view is supported by the results of the present study. The overall connectivity pattern revealed by DCM was the same for EPI and EPIK. DCM analyses for both imaging sequences identified a bidirectional model, involving task-related modulations of connections between visual and motor areas as the best model. Yet, the hypothesis that the higher spatial resolution provided by EPIK might allow a more consistent and more reliable estimation of coupling parameters was confirmed for one of the connections investigated. Overall, parameter estimates based on EPIK were at least as robust as those observed with EPI. Moreover, significantly more robust parameter estimates were found from the EPIK data for intrinsic couplings from the left motor cortex to the visual cortex in the right hemisphere. This pattern is consistent with the findings from our analysis of BOLD amplitudes where EPIK, as compared to EPI, revealed stronger BOLD signals in the right occipital lobe, suggesting that brain regions that benefit from a higher spatial resolution due to EPIK with regard to BOLD amplitudes will also allow a more reliable modelling of coupling parameters.

The effects of local susceptibility gradients, which could effectively push the gradient echoes in the EPI train out of the sampling window^27^, need to be considered in EPIK as it shortens the total duration of the sampling window. Employing the formula given by Deichmann *et al*., the echo time modified by the local susceptibility gradients, TE’ is given as follows:

$$\begin{array}{c}Q=1-\gamma \cdot \Delta t/(2\pi )\cdot FOV\cdot {G}_{SP}\\ TE\mbox{'}=TE/Q,\end{array}$$

where *𝛾*, *𝛥t* and *G*_*SP*_ are the gyromagnetic ratio, echo spacing time and local susceptibility gradient, respectively. The formula suggests that in certain brain regions where the *G*_*SP*_ has a positive value, the effective echo time, TE’, is presented as an increased value of the nominal TE and hence, it leads to signal drop out. A typical case of this can be seen in the frontal region. As can be seen from the formula, for a given positive *G*_*SP*_, this TE change becomes bigger when echo spacing time (*𝛥t*) is bigger. As calculated in this work, since the echo spacing of EPIK is shorter than that of EPI, it is expected that the TE’ of EPIK is always smaller than that of EPI in such brain regions and hence, has a reduced signal drop out. This has been experimentally verified by the *in vivo* data (see Fig. 3).

Moreover, although the effective echo spacing is shorter in EPIK, as its matrix size (96) in this work is larger than that of EPI (64), the total sampling window of EPIK is not shorter. Considering the TE (30 ms) and the effective echo spacing of each method, the sampling window of EPI is given as 13.68 ms (i.e. 30 ms − 0.510 ms × 32) < TE < 46.32 ms (i.e. 30 ms + 0.510 ms × 32) and for EPIK is given as 8.64 ms (i.e. 30 ms - 0.445 ms × 48) < TE < 51.36 ms (i.e. 30 ms + 0.445 ms × 48), respectively. This calculation indicates that the employed EPIK sequence has a wider sampling window size (10.08 ms wider) and therefore, was able to cover more brain regions affected by various degrees of susceptibility gradients (*G*_*SP*_) than the employed EPI.

The present work employed non-accelerated EPI and EPIK to avoid the complicating effects of acceleration techniques. The use of acceleration techniques (e.g. parallel imaging, partial Fourier or multi-band techniques) in EPIK is as straightforward as in EPI^6,7,28^. As the acquisition efficiency increases by combining the acceleration approaches introduced above, the use of EPIK method could potentially be extended to fMRI studies aiming at columnar resolution and/or sub-second image acquisition. Importantly, the advantages arising from these acceleration techniques are expected to accrue equally for both EPI and EPIK, as indeed are the disadvantages. However, the application of EPIK for the task-based BOLD and the resulting connectivity measurement have not been performed yet. Therefore, this work is focused on the performance evaluation of both EPI and EPIK methods without any acceleration conditions. Both EPI and EPIK would benefit equally from the use of acceleration techniques such as multiband excitation.

Furthermore, in high-resolution EPI, TE will be necessarily increased for a given set of parameters and therefore EPI will underperform compared to EPIK. That is, under these circumstances, a longer acquisition window accumulates more errors caused by field inhomogeneities, which may lead to unacceptable image quality for high-resolution applications. The claim of higher spatial resolution for EPIK may, at first sight, seem contradictory for a method where less time is spent acquiring the peripheral k-space lines. This is, however, manifestly not the case. First, the PSF of EPIK was shown to be sharper than that of EPI, which suggests that EPIK will have less blurring artefacts and thus a better spatial resolution than EPI^4^. This has been verified by the PSF simulation for the cases when time-series data are stationary as well as when dynamically varying. This, in turn, implies that EPIK is superior to EPI in localising the functional signals. Next, considering the fact that the haemodynamic response changes quite slowly, it is not always necessary to update the peripheral k-space at a high rate for high-resolution mapping. As long as the peripheral k-space is updated more quickly than the haemodynamic response changes, the spatial extent of the functional signals will be faithfully captured. In EPIK, the peripheral k-space update rate is given by 1/(TR · number of shots). In our study, it is 1/6.6 s (i.e. 0.15 Hz), which is much quicker than the period of haemodynamic response changes (1/32 s ≃ 0.03 Hz). The peripheral k-space update rate of EPIK in the present study also suggests the use of EPIK for resting-state fMRI as its target functional signals fluctuate very smoothly (<0.1 Hz)^29^. In event-related fMRI, the haemodynamic response changes are not, in general, as smooth as in blocked paradigms. However, if a shorter TR is used to further increase the peripheral update rate, then a utility of EPIK for event-related fMRI can be also expected. Nevertheless, the application of EPIK for resting-state and event-related fMRI needs to be carefully explored in future studies.

## Conclusions

In order to evaluate the performance of EPIK in terms of BOLD detection and functional connectivity, the application of EPIK to a visually-guided finger-tapping paradigm has been demonstrated here at 3 T. The performance of the EPIK method was evaluated by inspecting the quality of the reconstructed images, as well as the functional quantities obtained, and comparisons were made with those from a standard, single-shot EPI method. The reconstructed images from a representative fMRI session verified that EPIK yielded higher resolution images than EPI, with a slight decrease in the number of slices acquired. Furthermore, a substantial reduction of geometric distortions, arising from susceptibility differences, was observed in EPIK compared to EPI. The analysis of functional data revealed that when directly compared to EPI, EPIK yielded stronger BOLD responses in the primary and supplementary motor areas as well as in the cerebellar regions and in the visual cortex. This indicates that EPIK was especially sensitive to BOLD signal changes in the core regions of the network. The analysis of effective functional connectivity between the visual and motor areas suggests that EPIK can be also used in functional connectivity studies and may allow for a more robust estimation of coupling parameters. Overall, it has been shown that EPIK performs better than EPI and can be one of the methods of choice for fMRI.

## Supplementary information

Appendix
