# Supplementary material for: Evaluating the Utility of EPIK in a Finger Tapping fMRI Experiment using BOLD Detection and Effective Connectivity
Source: Sci Rep. 2019 Jul 29;9:10978. doi: 10.1038/s41598-019-47341-y (PMC6662889; doi:10.1038/s41598-019-47341-y)
Supplement: Supplementary file 1 — Appendix [file 41598_2019_47341_MOESM1_ESM.docx]

**Evaluating the Utility of EPIK in a Finger Tapping fMRI Experiment**

**using BOLD Detection and Effective Connectivity**

Seong Dae Yun^1^, Ralph Weidner^2^, Peter H. Weiss^2^ & N. Jon Shah^1,3,4,5^*

Underlined authors contributed equally to this work.

**Appendix: comparison of image resolution and geometric distortions**

In this appendix, the performance of EPIK in terms of image resolution and geometric distortions is further evaluated using the same imaging condition as EPI. For each imaging method (EPI and EPIK), two voxel sizes (3.13 × 3.13 mm^2^ and 2.08 × 2.08 mm^2^) were used and hence, in total, four different protocols were employed as shown in Supplementary Fig. S1. The TE (45ms) was the same for every protocol. The purpose of the relatively long TE was to acquire EPI data with a higher resolution (2.08 × 2.08 mm^2^; matrix size of 96 × 96) without using any in-plane acceleration technique; for EPIK, it is possible to acquire the higher resolution data without any acceleration technique as demonstrated by the present work. Moreover, the echo spacing of each phase encoding gradient was also kept identical at 740 ms for all protocols.

The four protocols outlined above were employed on a Magnetom Prisma 3T MRI scanner (Siemens, Erlangen, Germany) with a 20-channel phased array coil from the manufacturer. For the acquisition of *in vivo* data, another healthy male volunteer - not the same subject used for our main experiments - was scanned. Two representative slices were chosen from the acquired data to demonstrate the performance of EPIK in terms of the enhanced spatial resolution and the robustness against the geometric distortions caused by the susceptibility differences. Supplementary Fig. S1 depicts the obtained images for the four different protocols and for the two different slice locations. From the leftmost to the rightmost column, images from the four protocols are presented, while from the top to bottom row, images from different slice location are displayed. For both slice locations, the EPIK images reveal reduced geometric distortions around the frontal lobe when compared to the EPI images. This reduced geometric distortion is also clear when comparing the EPI and EPIK scan with a different matrix size (i.e. EPI with 3.13 × 3.13 mm^2^ vs. EPIK with 2.08 × 2.08 mm^2^). It is also clearly observed that images with a smaller voxel (2.08 × 2.08 mm^2^) size have better spatial resolution than those with a larger voxel size (3.13 × 3.13 mm^2^). However, even for the same voxel size, the EPIK scans (see the last column) exhibit enhanced spatial resolution compared to EPI scans (see the third column), particularly for the regions marked by arrows. Here, enhance spatial presentation of anatomical structures (e.g. gyri) can be observed in EPIK.

**
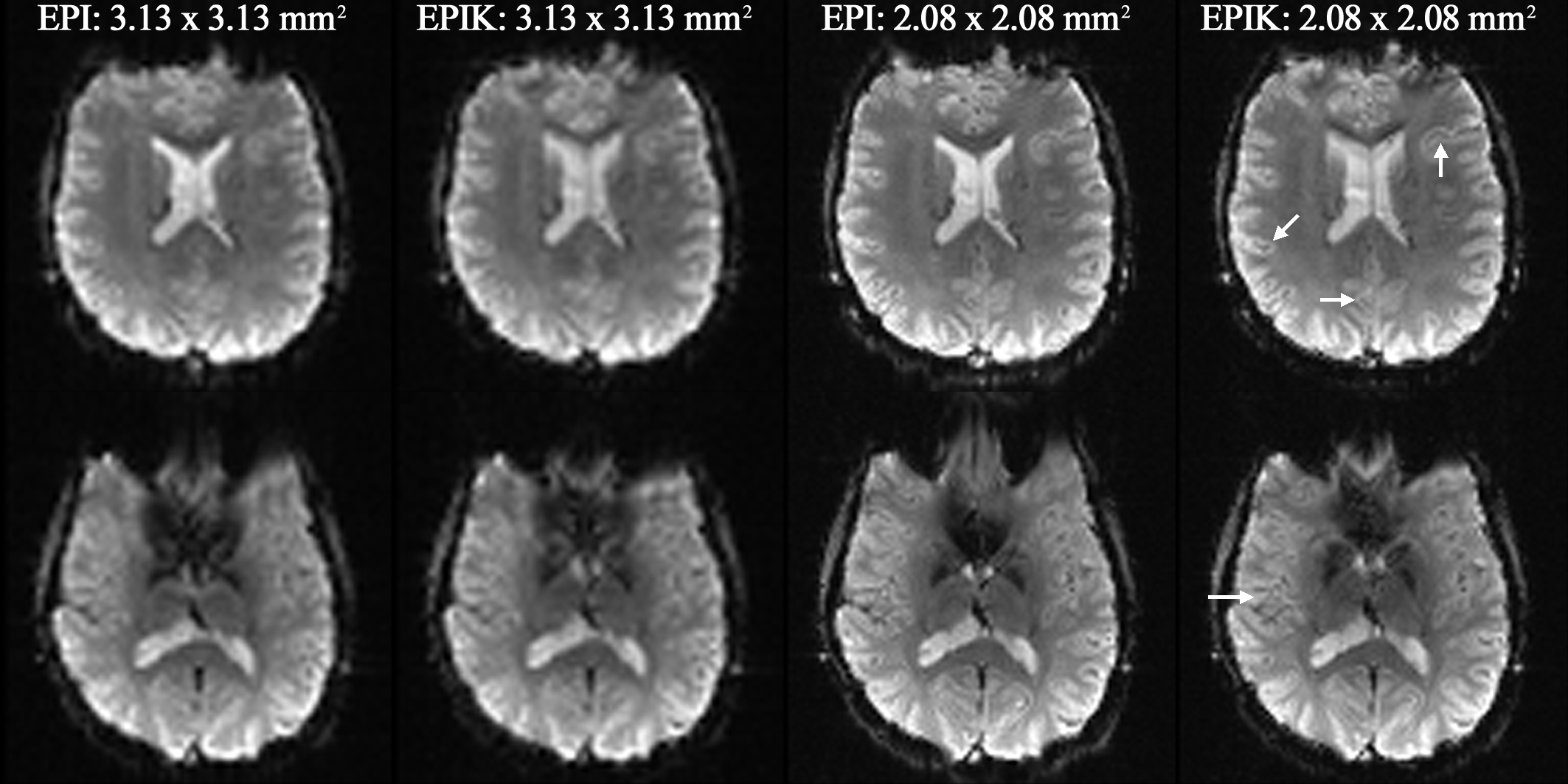
**

**Supplementary Figure S1** Reconstructed images acquired with EPI and EPIK. From the leftmost to rightmost column, images from four different protocols are presented: i) EPI (3.13 × 3.13 mm^2^), ii) EPIK (3.13 × 3.13 mm^2^), iii) EPI (2.08 × 2.08 mm^2^) and iv) EPIK (2.08 × 2.08 mm^2^). The top row and the bottom row depict different slice locations. EPIK images have reduced geometric distortions around the frontal lobe when compared to EPI images. In addition, particularly for the regions marked by arrows, the EPIK image (last column) reveals enhanced spatial presentation of anatomical structures than the EPI image (third column).
